# Activation of phagocytic activity in astrocytes by reduced expression of the inflammasome component ASC and its implication in a mouse model of Alzheimer disease

**DOI:** 10.1186/s12974-016-0477-y

**Published:** 2016-01-27

**Authors:** Julien Couturier, Ilie-Cosmin Stancu, Olivier Schakman, Nathalie Pierrot, François Huaux, Pascal Kienlen-Campard, Ilse Dewachter, Jean-Noël Octave

**Affiliations:** Université catholique de Louvain, Avenue Hippocrate 54, B1.5410, B-1200 Brussels, Belgium; Institute of Neuroscience, Université catholique de Louvain, Avenue Hippocrate 54, B1.5410, B-1200 Brussels, Belgium; Louvain Centre for Toxicology and Applied Pharmacology (LTAP), Université catholique de Louvain, Brussels, Belgium

**Keywords:** Alzheimer disease, Inflammasome, ASC, Astrocytes, CCL3, amyloid-β, Phagocytosis

## Abstract

**Background:**

The proinflammatory cytokine interleukin-1β (IL-1β) is overexpressed in Alzheimer disease (AD) as a key regulator of neuroinflammation. Amyloid-β (Aβ) peptide triggers activation of inflammasomes, protein complexes responsible for IL-1β maturation in microglial cells. Downregulation of NALP3 (NACHT, LRR, and PYD domains-containing protein 3) inflammasome has been shown to decrease amyloid load and rescue cognitive deficits in a mouse model of AD. Whereas activation of inflammasome in microglial cells has been described in AD, no data are currently available concerning activation of inflammasome in astrocytes, although they are involved in inflammatory response and phagocytosis. Here, by targeting the inflammasome adaptor protein ASC (apoptosis-associated speck-like protein containing a CARD domain), we investigated the influence of activation of the inflammasome on the phagocytic activity of astrocytes.

**Methods:**

We used an ASC knockout mouse model, as ASC is a central protein in the inflammasome, acting as an adaptor and stabilizer of the complex and thus critical for its activation. Lipopolysaccharide (LPS)-primed primary cultures of astrocytes from newborn mice were utilized to evaluate Aβ-induced inflammasome activation by measuring IL-1β release by ECLIA (electro-chemiluminescence immunoassay). Phagocytosis efficiency was measured by incorporation of bioparticles, and the release of the chemokine CCL3 (C-C motif ligand 3) was measured by ECLIA. ASC mice were crossbred with 5xFAD (familial Alzheimer disease) mice and tested for spatial reference memory using the Morris water maze (MWM) at 7–8 months of age. Amyloid load and CCL3 were quantified by thioflavine S staining and quantitative real-time polymerase chain reaction (qRT-PCR), respectively.

**Results:**

Cultured astrocytes primed with LPS and treated with Aβ showed an ASC-dependent production of IL-1β resulting from inflammasome activation mediated by Aβ phagocytosis and cathepsin B enzymatic activity. ASC+/− astrocytes displayed a higher phagocytic activity as compared to ASC+/+ and ASC −/− cells, resulting from a higher release of the chemokine CCL3. A significant decrease in amyloid load was measured in the brain of 7–8-month-old 5xFAD mice carrying the ASC +/− genotype, correlated with an increase in CCL3 gene expression. In addition, the ASC +/− genotype rescued spatial reference memory deficits observed in 5xFAD mice.

**Conclusions:**

Our results demonstrate that Aβ is able to activate astrocytic inflammasome. Downregulation of inflammasome activity increases phagocytosis in astrocytes due to the release of CCL3. This could explain why downregulation of inflammasome activity decreases amyloid load and rescues memory deficits in a mouse model of AD.

**Electronic supplementary material:**

The online version of this article (doi:10.1186/s12974-016-0477-y) contains supplementary material, which is available to authorized users.

## Background

Alzheimer disease (AD), the most common cause of dementia in the elderly, is characterized by alteration of cognitive functions, coexistence in the brain of senile plaques and neurofibrillary tangles, and neuronal loss [1].

Over the past decade, deposition of amyloid-β (Aβ) peptide in senile plaques has been shown to be the driving force of a strong and chronic inflammatory response [2, 3], characterized by the release of proinflammatory cytokines by reactive microglial cells and astrocytes surrounding amyloid deposits. This neuroinflammation plays a critical role in the pathogenesis of AD, by inducing neuronal toxicity leading to cognitive deficits [4, 5], but also contributes to protective mechanisms, since glial reactivity increases phagocytosis and clearance of Aβ deposits [6].

Interleukin-1β (IL-1β) is detected in astrocytes and microglial cells in the brains of AD patients as well as in animal models of AD [7–12]. Since IL-1β lacks a signal peptide, it is produced as the inactive precursor pro-IL-1β and requires processing by the cysteine protease caspase-1 to be active [13, 14]. Cytosolic multiprotein complexes called “inflammasomes” tightly control the activity of caspase-1. The inflammasome is an oligomeric protein complex organized as a tripartite structure: (1) a cytosolic danger sensor from the NLR (NOD-like receptor) family such as NALP3 (NACHT, LRR, and PYD domains-containing protein 3) also known as NLRP3 (NOD-like receptor family, pyrin domain containing 3) and NALP1 or IPAF (ice protease-activating factor), (2) the proteolytic effector caspase-1, and (3) ASC (apoptosis-associated speck-like protein containing a CARD domain), an adaptor protein needed to recruit NLR and to stabilize caspase-1/NLR complexes [15].

It was recently demonstrated that inflammasome containing NALP3 can be activated by Aβ peptide in vitro, leading to inflammation and tissue damage [7]. Moreover, NALP3 deficiency in the APP/PS1 AD mouse model results in a decrease in Aβ deposition and rescue of memory deficits [16]. Although most of the studies focused so far on the possible implication of microglial inflammasome in AD, recent studies suggest that astrocytic inflammasome can also play a role. Downregulation of astrocytic IPAF inflammasome reduces Aβ42 generation by primary neurons, and expressions of IPAF and ASC are significantly increased in a subgroup of sporadic AD patients [17].

In the present study, we investigated the role of ASC-mediated activation of the inflammasome on phagocytic activity of astrocytes and its implication in a mouse model of AD. We found that ASC was essential for IL-1β release from primary astrocytes exposed to Aβ42. Interestingly, we showed that the heterozygous expression of ASC in ASC+/− astrocytes was responsible for an increased phagocytic activity linked to the release of CCL3 (C-C motif ligand 3). In vivo, we showed, in the 5xFAD (familial Alzheimer disease) mouse model, that heterozygous ASC expression led to a decrease in amyloid load with a rescue of long-term spatial memory measured in the Morris water maze (MWM).

## Methods

### Animals

All animal procedures used in the study were carried out in accordance with institutional and European guidelines as certified by Animal Ethics Committee.

*ASC* knockout mice on the C57Bl6/J genetic background [18] were purchased from Charles River Laboratory (Brussels, Belgium).

We used 5xFAD transgenic mice [19] overexpressing mutant human APP695 carrying EOFAD mutations K670N/M671L (Swedish) + I716V (Florida) + V717I (London) and mutant human PS1 harboring 2 EOFAD mutations (M146L and L286V) driven by the thymocyte differentiation antigen 1 (ThyI) promoter (JAX Mice and Services, Bar Harbor, ME, USA).

We crossbred hemizygous 5xFAD mice with ASC mice. Experiments on 5xFAD ASC mice and age-matched control were performed at 7–8 months of age.

All mice were genotyped by PCR analysis of tail biopsies. Animals were housed on a 12-h light/dark cycle in standard animal care facilities with access to food and water ad libitum.

### Primary cultures of astrocytes from newborn mice and treatments

All cell culture reagents were purchased from Invitrogen (Carlsbad, CA). Primary glial cultures were prepared from newborn ASC mice (P0-1). Newborn mice were euthanized by decapitation and genotyped. The brains were removed, and the cortico-hippocampal regions were dissected in phosphate-buffered saline. Cells were then mechanically dissociated in Dulbecco’s Modified Eagle’s Medium (DMEM) (GlutaMAX)/penicillin-streptomycin (PS, 50 mg/ml), and the supernatant was collected and centrifuged at 280×*g* for 5 min. Cells were suspended in DMEM-GlutaMAX/PS supplemented with 10 % fetal bovine serum (FBS) and PS. Cells were seeded in 72-cm^2^ culture flasks and then incubated in a humidified 5 % CO_2_ atmosphere at 37 °C. The medium was changed every 5 days, and cultures were maintained for 15 days.

At day 15, non-astroglial cells such as microglia and oligodendrocytes were removed by shaking 5 h at 200 rpm/37 °C in an orbital shaker. Remaining adherent cells were then trypsinized (trypsin-EDTA 0.05 %) and plated at 10^4^ cells/cm^2^ in appropriate non-coated wells. Immunofluorescent staining using antibodies against CD68 (microglia) (AbdSerotec) and GFAP (glial fibrillary acidic protein for astrocytes) (Dako) revealed that >98 % of cells were astrocytes.

Astroglial cultures were used at day 4 after subculture. Cells were primed with lipopolysaccharide (LPS) from *Escherichia coli* 026:B6 (Sigma-Aldrich), at 1 μg/ml for 3 h, washed with fresh medium and then treated for 3 h with 20 μM nigericin or 10 μM Aβ42 (Bachem), previously suspended in milliQ water, incubated 72 h at 37 °C for aggregation [20], and diluted in serum-free medium. Cathepsin B inhibitor Ca074 at 25 μM, or 10 μM cytochalasin D, was added 15 min before Aβ42.

### RNA extraction, quantitative real-time PCR, and PCR

Total RNA extraction was performed according to TriPure Isolation Reagent manufacturer’s instructions (Roche). Reverse transcription was carried out from 1 μg of extracted RNA resuspended in DEPC-treated water, using the iScript cDNA synthesis Kit (Bio-Rad). Quantitative real-time polymerase chain reaction (qRT-PCR) conducted with the iCycler IQ^TM^ multicolor Real-Time PCR Detection System (Bio-Rad) was carried out from 2 ng of cDNA template, specific primers at 0.3 μM and the IQ^TM^ SYBR® Supermix 1x. Murine primers (Sigma-Aldrich) used for *CCL3*, *GAPDH* (glyceraldehyde 3-phosphate dehydrogenase) and for *IL-1β* are presented in the Table 1.

**Table 1:** List of primers used and size of PCR products

Gene	Primer sequences	Size (bp)
*CCL3*	Forward: 5′-ATGAAGGTCTCCACCACTGC-3′	196
	Reverse: 5′-TCAGGAAAATGACACCTGGCT-3′	
*IL-1β*	Forward: 5′-ATGAAGGGCTGCTTCCAAAC-3′	186
	Reverse: 5′-GAAGGTGCTCATGTCCTCATC-3′	
*GAPDH*	Forward: 5′-ACCCAGAAGACTGTGGATGG-3′	172
	Reverse: 5′-ACACATTGGGGGTAGGAACA-3′	
*ASC*	Forward: 5′-CTAGTTTGCTGGGGAAAGAAC-3′	394
	Reverse: 5′-CTAAGCACAGTCATTGTGAGCTC-3′	

The PCR protocol consisted of 40 amplification cycles with the following steps: 95 °C for 30 s, 60 °C for 45 s, and 79 °C for 15 s. The result for each sample was normalized with the relative expression of *GAPDH*, and relative amplifications were calculated by the 2-ΔΔCt method. For standard PCR, 10 μl was used and amplification was performed using 0.125 μl of *Taq* DNA Polymerase (Thermo Fisher Scientific) and murine primers for *ASC* (Table 1). Cycling was performed in a TProfessional Thermocycler (Biometra), and the amplification protocol consisted of 35 amplification cycles with the following steps: 95 °C for 30 s, 57 °C for 1 min, and 72 °C for 2 min. Aliquots of 8 μl of each PCR products were loaded on a 1.2 % agarose gel with the Midori Green Advanced DNA Stain (Nippon Genetics Europe GmbH) as nucleic acid stain.

### ECLIA

IL-1β and CCL3 were quantified in culture media from astrocytes by ECLIA (electro-chemiluminescence immunoassay) on a MSD SECTOR^TM^ Imager 2400 (Meso Scale Discovery), following manufacturer’s instructions. The lower limit of detection (LLOD) for IL-1β was 0.6 pg/ml, whereas no LLOD was given by the manufacturer for CCL3 as it was a prototype kit.

### Phagocytosis assay and CCL3 neutralization

Phagocytosis assay was performed following supplier’s instructions with pHrodo™ Red Zymosan A BioParticles® (Life Technologies) conjugate for phagocytosis. Briefly, LPS-primed astrocytes plated in 96 wells were treated with 10 μM Aβ42 for 3 h. Bioparticles (0.5 mg/ml) were added 1 h after Aβ42. CCL3-neutralizing antibodies (R&D systems) were added 15 min before bioparticles at a concentration of 0.1 μg/ml, corresponding to the manufacturer’s estimated ND50 range for CCL3-detected levels in our model. Primary astrocytes were also exposed to mouse recombinant CCL3 (R&D systems) at concentrations ranging from 1.5 to 10 ng/ml, to evaluate a specific effect of the chemokine in astrocytic phagocytic activity. After 2 h of incubation with bioparticles, pictures were taken using a digital inverted fluorescence microscope (EVOS-xl; Life Technologies) with a ×4 lens. An ECLIA for CCL3 was performed to evaluate neutralization efficacy after LPS priming and Aβ42 treatment.

### Thioflavine S staining

Mice were transcardially perfused with ice-cold PBS to remove blood. Brains were extracted, and the right cerebral hemisphere was fixed by immersion in a 4 % paraformaldehyde solution for 24 h at 4 °C. Forty micrometer-sagittal sections were cut on a vibrating HM650V microtome (Thermo Scientific) and were preserved in PBS/azide 0.1 %. Staining with thioflavin-S (ThioS; Sigma-Aldrich), a specific β-sheet strand intercalant, was performed on brain sections as described previously [21]. Image acquisition was performed using a digital inverted fluorescence microscope (EVOS-xl; Life Technologies) with a ×4 lens. Plaques were quantified using Image J software (U.S. National Institutes of Health, Bethesda, MD, USA) by measuring the area of ThioS staining in a well-defined selected area of the hippocampus.

### Behavioral analysis

The MWM with a mild learning paradigm was used for studying spatial learning and memory. The MWM was essentially performed as previously described [21]. Age-matched mice (7 months old) were used. A circular polypropylene pool (113 cm in diameter), filled with white-opaque water (24 ± 1 °C) for hiding the submerged escape platform (9.5 cm diameter), was used for testing. Mice were trained to locate the hidden platform in three trials during one training day with an inter-trial interval of 1 h. Training over six consecutive days was performed. Twenty-four hours after the last training session on day 7, the platform was removed for a 60-s probe trial. Mouse behavior in the pool was videotaped and tracked using Ethovision camera and software (EthoVision 6.1 Noldus, Wageningen, The Netherlands).

### Statistical analysis

The number of samples or animals is specified in the caption for each experiments. Results are expressed as the mean ± SEM. Statistical analysis was performed by either one-way ANOVA with post Bonferroni multiple comparison test or one-way ANOVA for repeated measures for the training in MWM. All analyses were performed using GraphPad Prism software (GraphPad Software Inc). Statistical significance was defined as *P* < 0.05.

## Results

### IL-1β is released by astrocytes in a cathepsin B-dependent manner

IL-1β can be produced and released by several cell types, including activated monocytes and macrophages, as well as by microglial cells and astrocytes [17, 22, 23]. Recent studies have shown that LPS-primed primary microglial cells are able to produce IL-1β after activation of inflammasomes by oligomeric or fibrillar Aβ treatment [7, 24].

**Fig. 1:**
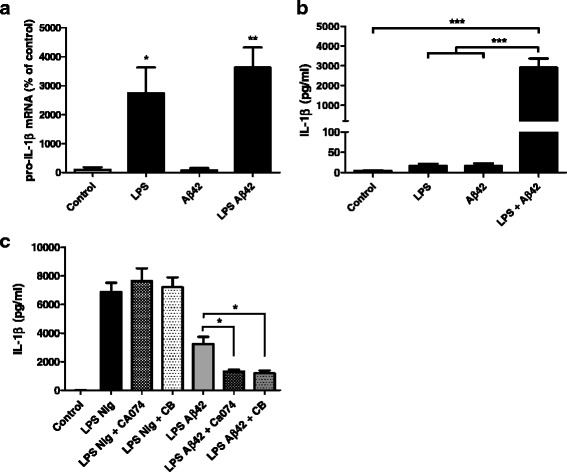
Aβ42 induces cathepsin B-dependent IL-1β release in LPS-primed astrocytes. Astrocytes primed with 1 μg/ml LPS for 3 h were treated with 10 μM Aβ42 for 3 h. *Pro-IL-1β* expression was determined by qRT-PCR (**a**) (*n* = 4), and IL-1β was measured by ECLIA in culture supernatants (**b**) (*Control* and *LPS n* = 7 in duplicate; *Aβ42* and *LPS Aβ42 n* = 5 in duplicate). Primed astrocytes were exposed to 20 μM nigericin or 10 μM Aβ42, with 25 μM Ca074 or 10 μM cytochalasin D ,and IL-1β was measured by ECLIA in culture supernatants (**c**) (*Control* and *LPS Nig n* = 7; all others *n* = 4). Results are the mean ± SEM, and statistical differences were determined using an ANOVA analysis and post Bonferroni multiple comparison test. ***P < 0.001, **P < 0.01, and *P < 0.05

To assess the ability of astrocytes to produce and release IL-1β when treated with Aβ42, we used a model of primary astrocytes from P0-1 newborn mice primed with LPS, a TLR (Toll-like receptor) agonist, to induce a robust induction of transcription of the *pro-IL-1β*, as it is not constitutively expressed. Results of qRT-PCR showed a robust induction of *pro-IL-1β* after 3 h priming with 1 μg/ml LPS, with or without Aβ42, but Aβ42 alone did not induce any expression of *pro-IL-1β* (Fig. 1a), highlighting the need of LPS priming. We confirmed that IL-1β was secreted by primary astrocytes primed for 3 h with 1 μg/ml LPS and treated with 10 μM Aβ42 for 3 h, whereas release of IL-1β was minimal with LPS and Aβ42 alone (Fig. 1b).

In microglial cells, the NALP3 inflammasome is known to be activated by various toxins like nigericin, inducing a K+ efflux, and by phagocytosis of several crystals or aggregates (including Aβ). As astrocytes are known to phagocytose Aβ [25], we investigated whether Aβ-induced release of IL-1β was dependent on phagocytosis and subsequent release of cathepsin B from phagolysosome.

We incubated astrocytes with 25 μM Ca074, a cathepsin B inhibitor, or with 10 μM cytochalasin D, 15 min before and during Aβ treatment. We found that both cytochalasin D and Ca074 were able to attenuate Aβ-induced release of IL-1β, whereas no effect was observed on astrocytes treated with 20 μM nigericin (Fig. 1c), indicating that the induction of IL-1β by Aβ42 requires phagocytosis and lysosomal rupture with cathepsin B leakage in the cytosol. These observations demonstrate that phagocytosis by astrocytes and lysosomal rupture with cathepsin B leakage in the cytosol play an important role in the Aβ-induced maturation of IL-1β.

### IL-1β release is ASC-dependent

**Fig. 2:**
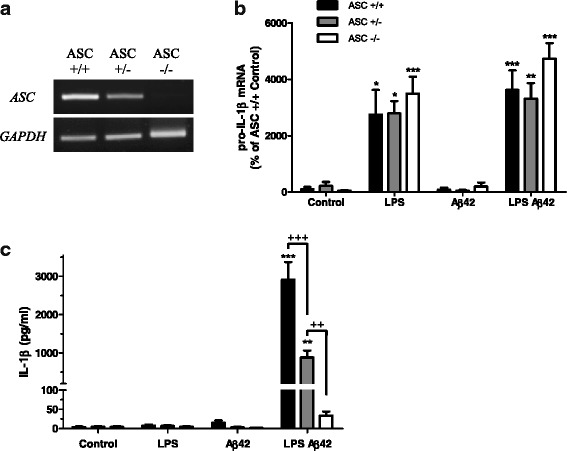
Aβ42-induced IL-1β release in LPS-primed astrocytes is ASC dependent. RT-PCR with specific primers set for murine *ASC* and *GAPDH* was performed on cDNA from astrocytes primed with 1 μg/ml LPS for 3 h (**a**). *Pro-IL-1β* expression was quantified by qRT-PCR on cDNA from astrocytes primed with 1 μg/ml LPS for 3 h and/or treated with 10 μM Aβ42 for 3 h (**b**) (*n* = 4). The concentration of IL-1β in culture supernatants was then measured by ECLIA (**c**) (*Control n* = 5 in duplicate; *LPS Aβ42 n* = 4 in duplicate for all genotypes). Results are the mean ± SEM, and statistical differences were determined using an ANOVA analysis and post Bonferroni multiple comparison test. ***P < 0.001, **P < 0.01, and *P < 0.05 vs ASC+/+ control (**b**). ***P < 0.001 and **P < 0.01 vs respective controls; ^+++^ *P* < 0.001 and ^++^ *P* < 0.01 (**c**)

To evaluate ASC involvement in IL-1β production by astrocytes, we used astroglial primary cultures from P0-1 newborn mice primed with LPS. As expected, *ASC* expression after LPS priming was totally abolished in ASC−/− astrocytes and showed an intermediate level in ASC+/− compared to ASC+/+ (Fig. 2a). High and comparable expressions of *pro-IL-1β* were observed in LPS primed or in LPS + Aβ42 astrocytes in all genotypes, but still, no induction was found with Aβ42 alone (Fig. 2b). Taken together, these results demonstrate that induction of *pro-IL-1β* is independent of ASC.

Release of mature IL-1β was then quantified by ECLIA in astrocytes primed 3 h with 1 μg/ml LPS and treated 3 h with 10 μM Aβ42. Production of soluble IL-1β was significantly decreased in ASC+/− and abolished in ASC−/− astrocytes, indicating a specific requirement of ASC in Aβ-induced maturation and secretion of IL-1β (Fig. 2c).

### ASC heterozygous astrocytes are more prone to phagocytize

It is obvious that microglial cells are the immunocompetent cells of the central nervous system (CNS) that display common functions with macrophages, including phagocytosis. However, even if the role of astrocytes in phagocytic activities is less studied, some data suggest that they are competent phagocytes. Recent studies have shown an Aβ-induced phagocytosis of latex beads [25] as well as direct engulfment of Aβ [26].

We therefore investigated the phagocytic capacity of astrocytes expressing different levels of ASC. LPS-primed astrocytes were treated with 10 μM Aβ42 and a highly specific phagocytosis assay was applied. It consists in pH-sensitive-labeled yeast wall beads exhibiting fluorescence upon acidification of the particles as they are ingested.

**Fig. 3:**
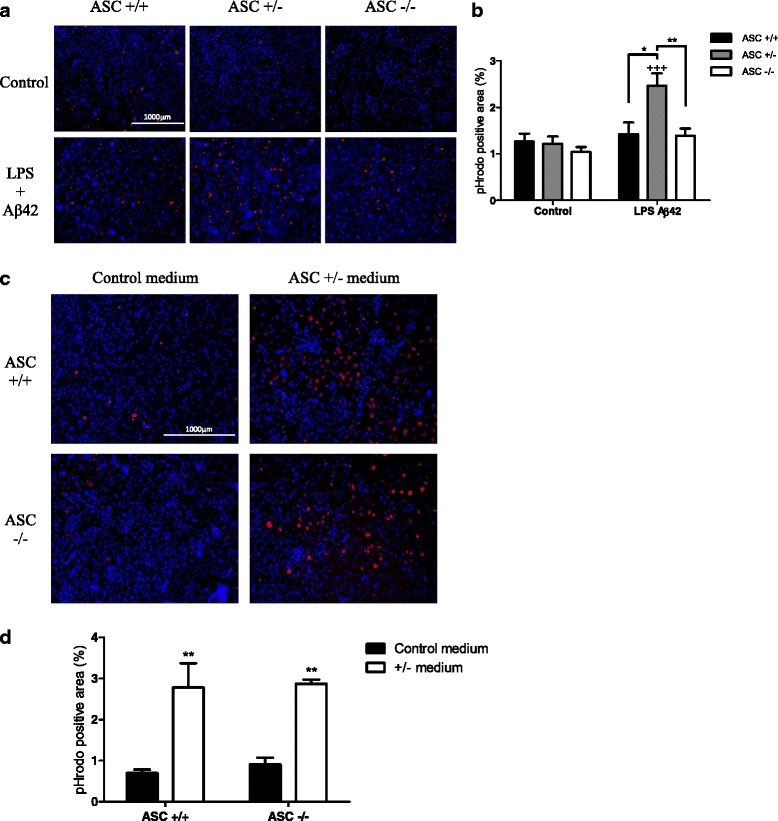
Phagocytosis is increased in ASC+/− LPS-primed astrocytes treated with Aβ42. Astrocytes primed with 1 μg/ml LPS for 3 h were treated with 10 μM Aβ42 for 3 h. Phagocytic capacity was assessed by adding pHrodo™ Red Zymosan Bioparticles® 2 h before visualization (**a**); pHrodo™ Red dye conjugates fluoresce brightly red only in phagolysosomes. Media from treated ASC+/− astrocytes were added to ASC+/+ and ASC−/− cells, and phagocytic capacity was assessed (**b**). Quantity of internalized bioparticles was evaluated by measuring fluorescence areas using image J (**b**, **d**). Hoechst staining was performed to evaluate cell density. Results are expressed as a percentage of pHrodo positive area and presented as the mean ± SEM (**a**, **b** *n* = 4 in duplicate; **c**, **d** *n* = 3 in duplicate). Statistical differences were determined using an ANOVA analysis, and post Bonferroni multiple comparison test. **P < 0.01 and *P < 0.05 vs ASC+/− LPS Aβ42 and ^+++^ *P* < 0.001 vs respective control (**b**), **P < 0.01 vs respective control (**d**)

A basal phagocytic activity was observed in control conditions. No significant increase was observed in LPS-primed ASC+/+ and ASC−/− astrocytes treated with Aβ42. But surprisingly, ASC+/− astrocytes showed a significant increase in their phagocytic capacity as shown using fluorescence microscopy (Fig. 3a) and after quantification of fluorescent areas (Fig. 3b).

These results raised the question whether such a difference could be due to an intrinsic property of ASC heterozygous astrocytes or to a change in the release of secreted factors. To answer that question, we exposed ASC+/+ and ASC−/− astrocytes to medium conditioned by ASC+/− astrocytes primed with LPS and treated with Aβ42. In both cases, we observed a strong increase of the phagocytic activity after exposure to ASC+/− medium (Fig. 3c, d), indicating that the modification in phagocytosis efficiency resulted from a change in the panel of released factors.

### CCL3 is involved in phagocytic activity of ASC heterozygous astrocytes

Chemokines play a critical role in phagocytic activity. Astrocytes are characterized by expression of a panel of chemokines including CCL3, which is expressed in murine astrocytes and binds receptors with a high affinity [27], inducing chemotaxis in the presence of low concentration of ligand [28]. Moreover, astrocytes express CCR1 and CCR5, two receptors known to bind CCL3 [29].

**Fig. 4:**
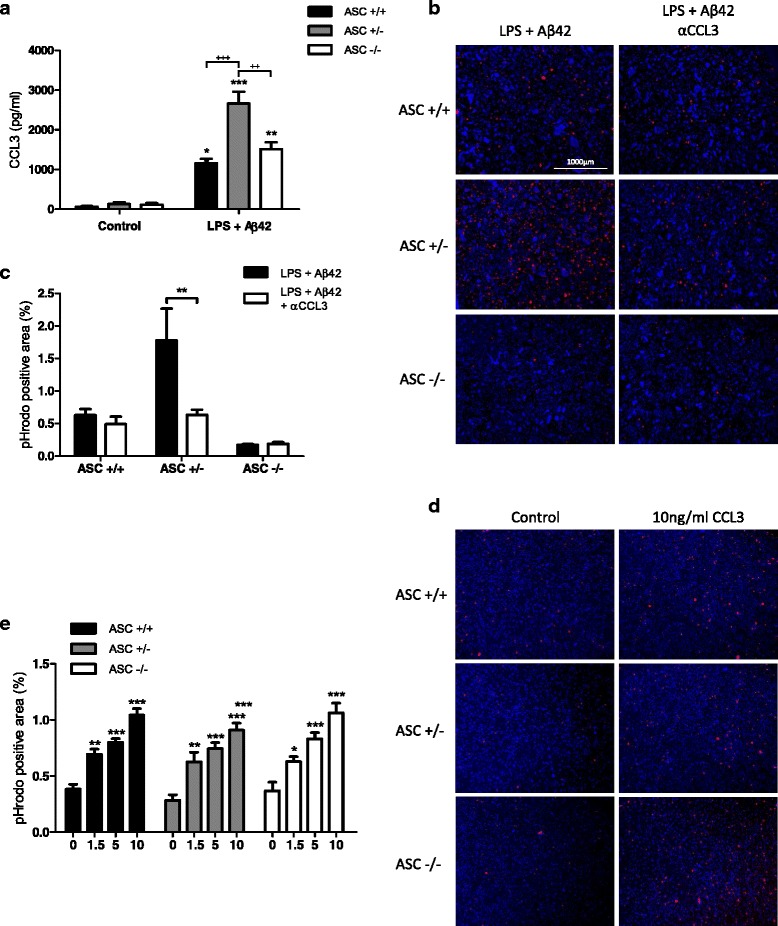
CCL3 release by astrocytes is ASC dependent and is critical for phagocytosis efficiency. Astrocytes primed with 1 μg/ml LPS for 3 h were treated with 10 μM Aβ42 for 3 h. The concentration of CCL3 in culture supernatants were then measured by ECLIA (**a**) (*Control n* = 4 in duplicate; *LPS Aβ42 n* = 7 in duplicate). Astrocytes were treated with 0.1 μg/ml of CCL3-neutralizing antibody 15 min before Aβ42 and phagocytic capacity was assessed by adding pHrodo™ Red Zymosan Bioparticles® 2 h before visualization (**b**, **c**). Primary astrocytes were treated with increasing concentrations of mouse recombinant CCL3 for 2 h during exposure to bioparticles. The amount of internalized bioparticles was evaluated by measuring fluorescence areas using image J (**c**, *n* = 4 in duplicate; **e**, *n* = 3 in triplicate). Results are expressed as a percentage of total area and are the mean ± SEM, and statistical differences were determined using an ANOVA analysis and post Bonferroni multiple comparison test: ***P < 0.001, **P < 0.01, and *P < 0.05 vs respective controls (**a**, **e**), ^+++^ *P* < 0.001 and ^++^ *P* < 0.01 (**a**); **P < 0.01 (**c**)

In astrocytes in which inflammasome was activated by Aβ42, we measured secreted CCL3 in the culture medium using ECLIA. We observed an increase in CCL3 released by astrocytes from each genotype compared to non-treated controls (Fig. 4a). Interestingly, ASC +/− astrocytes showed a significant higher level of CCL3 compared to ASC+/+ and ASC−/− astrocytes.

From these observations, we assessed phagocytic capacity of these astrocytes following CCL3 neutralization using a specific antibody. Neutralizing antibody was added 15 min before bioparticles, and the culture medium was recovered at the end of the experiment to measure CCL3 neutralization efficacy. In each case, anti-CCL3 antibody significantly attenuated CCL3 detectability in culture medium of astrocytes (Additional file 1; ASC+/+ 72 %, ASC+/− 64 %, and ASC−/− 40 %). Phagocytic activity was unaffected by CCL3 neutralization in ASC+/+ and ASC−/− astrocytes but a significant 64 % decrease was observed in ASC+/− cells (Fig. 4b, c). In order to evaluate CCL3 effect on astrocytic phagocytosis without the influence of other released factors, astrocytes were incubated in the presence of increasing concentrations of mouse recombinant CCL3 before measuring the incorporation of bioparticles. Results presented in Fig. 4d, e indicate that increasing concentrations of CCL3 induced significant increase in bioparticle incorporation by astrocytes, whatever their genotype. Altogether, these results indicate that increased secretion of CCL3 by ASC+/− astrocytes can activate their phagocytic activity.

### Decreased amyloid load in 5xFAD with heterozygous ASC expression is correlated with increased CCL3 expression

To evaluate, in an in vivo model, the relevance of our results obtained in vitro, we crossbred hemizygous 5xFAD with ASC knockout mice. We used 7–8 month mice as they display a strong hippocampal amyloid deposition and associated cognitive deficits. We focused on ASC+/− genotype that showed an optimal phagocytic activity in vitro.

**Fig. 5:**
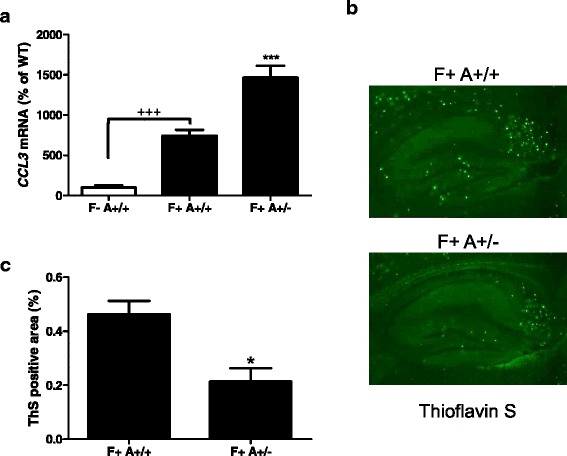
Increase in *CCL3* mRNA level is correlated with a decrease in amyloid load in ASC+/− 5xFAD 8-month-old mice. Expression of *CCL3* was determined by qRT-PCR on cDNA prepared from the hippocampus of 8-month-old WT and 5xFAD ASC mice. Statistical differences were determined using an ANOVA analysis and post Bonferroni multiple comparison test. ***P < 0.001 vs all others and ^+++^ *P* < 0.001 (**a**) (5 F− A+/+, 10 F+ A+/+, and 6 F+ A+/−). Thioflavin S staining was performed on sections from the hippocampus of 8-month-old WT and 5xFAD ASC mice (**b**) (7 F+ A+/+ and 4 F+ A+/−). Amyloid plaques were quantified using image J and are represented as the mean % of total area ± SEM. Significance was assessed using an ANOVA analysis and post Bonferroni multiple comparison test. *P < 0.05 vs all others (**c**)

From the in vitro results suggesting that CCL3 is critical for phagocytosis, we first assessed whether *CCL3* expression was changed in 5xFAD/ASC mice. As observed with primary culture of astrocytes, *CCL3* mRNA levels were increased in 5xFAD mice (F+ A+/+) compared to wild-type (WT, F-A+/+), but this increase was more important in the ASC heterozygous (F+ A+/−) genotype with an l00 % increase compared to F+ A+/+ (Fig. 5a).

In 5xFAD mice, amyloid deposits appear from 2 months of age in deep cortical layers and subiculum and increase with age in the cortex and hippocampus [19]. To assess the density of amyloid plaques, we stained sagittal sections of 7–8-month-old mice with thioflavin S and we quantified positive areas in the hippocampal region (Fig. 5b, c). We found that Aβ deposition was significantly reduced by about 60 % in F+ A+/− mice compared to F+ A+/+ (Fig. 5c). Quantification of the number of plaques showed the same results with a 45 % decrease in hippocampus of F+ A+/− mice (data not shown).

### Reduced ASC expression rescues memory deficits in 5xFAD mice

**Fig. 6:**
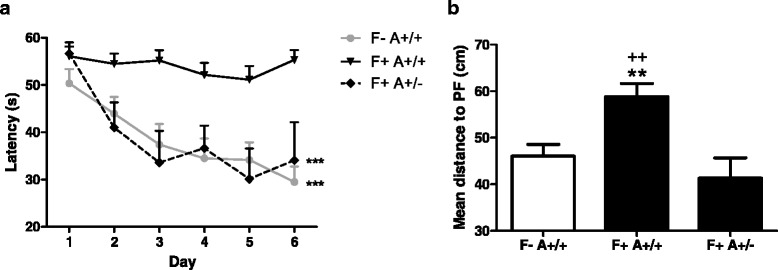
Memory deficits are prevented in 5xFAD mice with ASC+/− genotype. Hippocampal-dependent learning and long-term spatial memory were assessed in a modified Morris water maze paradigm. A protocol of three trials/day during 6 days was used as a training period for WT and 5xFAD ASC mice. Results are expressed as the mean latency to reach the platform ± SEM and analyzed by ANOVA for repeated measures, ***P < 0.001 vs F+ A+/+, (**a**). Probe trial was performed on day 7, and results are expressed as the mean distance to the platform (Gallagher proximity coefficient) ± SEM, and statistical differences were determined using a ANOVA analysis and post Bonferroni multiple comparison test, **P < 0,01 vs F− A+/+, ^++^ *P* < 0.01 vs F+ A+/− (**b**) (15 F− A+/+, 14 F+ A+/+, and 11 F+ A+/−)

Since we observed a decrease in amyloid deposition in the hippocampus of F+ A+/− mice, we assessed hippocampal-dependent spatial acquisition (training/learning) and reference memory (probe trial) in these mice using the MWM. Total path length during the probe trial was not modified meaning that mice did not display locomotor deficits (Additional file 2). 5xFAD mice failed to improve significantly the latency to reach the platform (PF) during the training period. However, this learning deficit was rescued in F+ A+/− mice, showing latency values equivalent to WT mice (Fig. 6a). F− A+/− did not show any difference with WT mice (data not shown).

We then tested the strength of long-term spatial memory by performing a probe trial, during which the PF has been removed when mice are swimming for 1 min. We used the Gallagher proximity measure (average distance in centimeters from the center of the platform location) [30] for the analysis of the probe trial, previously shown to be more sensitive to group differences [31]. Results showed a dramatic decline in long-term spatial memory of F+ A+/+ mice as the mean distance to the PF was significantly higher than WT mice (Fig. 6b). Nevertheless, F+ A+/− mice were largely protected from this decline, supporting performances observed during the training period.

## Discussion

Molecular platforms called inflammasomes are activated upon cellular infection or stress, triggering the maturation of proinflammatory cytokines such as pro-IL-1β to engage innate immune defenses [32]. It has been shown that aberrant inflammasome signaling contributes to pathology in a large number of infectious and autoimmune diseases [33]. However, their roles in CNS disorders have not been extensively studied, although recent literature suggests that inflammasomes are activated and participate to neurological diseases including infections, acute sterile brain injury, and chronic neurodegenerative diseases [34].

There is a high sensitivity of the CNS to IL-1β since multiple cerebral cell types express its receptor, which, upon activation, potentiates proliferation and activation of microglial cells and astrocytes [35, 36].

Previous studies have shown that the Aβ peptide of AD can activate the NALP3 inflammasome in microglial cells and induce release of inflammatory molecule IL-1β in vivo and in vitro [16, 24]. Aβ-mediated inflammasome activation in astrocytes remains, however, elusive.

In the present study, using primary culture of murine astrocytes, we show that LPS-primed cells are able to produce and release IL-1β under Aβ42 treatment, depending on ASC expression, as ASC−/− astrocytes did not produce significant amount of IL-1β. Activation of inflammasome in astrocytes was recently debated, from the absence of expression of NALP3, ASC, and caspase-1 in astrocytes [37] to colocalization of the GFAP astrocytic marker with NALP3 and ASC in a murine model of ALS [38]. We demonstrate here an activation of astrocytic ASC-dependent inflammasome by synthetic Aβ42, confirming thereby that, as microglial cells, astrocytes are able to produce IL-1β.

Phagocytosis of Aβ fibrils by macrophages or microglial cells was proposed to be the first step for the formation of the inflammasome complex, due to a leakage of cathepsin B from lysosomes into the cytosol [7]. Using cytochalasin D to inhibit phagocytosis or cathepsin B inhibitor, we show a strong attenuation of IL-1β release, indicating that Aβ42 triggers inflammasome activation through a lysosomal pathway, as is the case for microglial cells or macrophages. The mechanisms by which cathepsin B can activate the inflammasome are not clear. It was recently suggested that the Aβ-mediated release of cathepsin B leads to the degradation of the anti-inflammatory protein NLRP10 involved in inhibition of formation of the NALP3 inflammasome by interacting with ASC. Degradation of NLRP10 allows ASC-NALP3 interaction, caspase-1 activation, and IL-1β release [39].

As microglial cells, astrocytes express many potential phagocytic receptors in the CNS, like RAGE, CD36, or TLRs, known to have Aβ among their ligands [40]. But the role of astrocytes as phagocytic cells is less studied, particularly in AD. Addition of astrocytes on brain sections prepared from an AD mouse model decreases Aβ levels [26]. In APPswe/PS1dE9 mice, internalization of Aβ was observed in transplanted astrocytes [41]. These data confirm the role of astrocytes in amyloid clearance.

In the present study, we measured phagocytosis by astrocytes using fluorescent bioparticles. We show an increase in phagocytosis by ASC heterozygous astrocytes primed by LPS and treated with Aβ42. This increase was explained by the secretion of soluble factors since exposure of naive ASC+/+ or ASC−/− astrocytes to conditioned medium from ASC+/− cells induced an increase in phagocytosis.

Chemokines are critical inflammatory molecules inducing recruitment of responsive cells such as monocytes/macrophages, microglia, or astrocytes. Among them, CCL3, a CC chemokine, is known to bind receptors on murine astrocytes with a high affinity, stimulating chemotaxis. Three receptors are known to bind CCL3, and CCR1 and CCR5 are expressed at the membrane of astrocytes [29]. Therefore, we measured secretion of CCL3 in astrocytes in which inflammasome was activated by Aβ. Increase in CCL3 release was observed in all genotypes, with significantly higher levels with ASC+/− cells compared to ASC+/+ and ASC−/−. It was previously reported that astrocytes produce high amount of several chemokines and particularly CCL3 under stimulation by a mix of IL-1β and TNFα [42]. In our model, there was an inverse correlation between TNFα and IL-1β secretion (Additional file 3). Consequently, co-secretion of both TNFα and IL-1β is optimal in ASC+/− astrocytes, resulting in higher CCL3 production in these cells. It is important to notice that CCL2, another chemokine known to be produced by astrocytes, was expressed at a very high level in control condition and thus displayed a low rate of increase after cytokine stimulation. On the other hand, ASC downregulation using siRNA or shRNA has been shown to reduce MAPK pathway activation in the THP1 cell line [43] and to activate NF-κB in murine macrophages [44]. Modifications in activation of these pathways, known to be involved in the expression of cytokines and chemokines, could also contribute to modulation of the inflammatory environment.

Neutralization of released CCL3 by a specific neutralizing antibody induced a significant decrease in phagocytic activity of ASC+/− astrocytes. Moreover, treatment of astrocytes with mouse recombinant CCL3 alone induced a significant increase of astrocytic phagocytosis in each ASC genotype. Involvement of CCL3 in the phagocytic activity of various cell types, including macrophages, has been demonstrated. Macrophages stimulated with recombinant CCL3 displayed an increase phagocytic index against *Pseudomonas aeruginosa* [45]. Moreover, phagocytic activity of alveolar macrophages from CCL3−/− mice toward *Klebsiellapneumoniae* was dramatically decreased [46]. In vivo, CCL3 KO mice or WT mice treated with anti-CCL3 monoclonal antibodies were more prone to sepsis, suggesting a significant protective role for CCL3 [45].

We extended our analysis in vivo by crossing the very well-known 5xFAD mouse model with ASC deficient mice. Interestingly, we observed a very significant increase in *CCL3* mRNA levels in the brain of FAD/ASC heterozygous mice as compared to FAD/WT mice. In addition, a significant decrease in amyloid plaques was observed in FAD/ASC heterozygous mice at 7–8 months of age, with a concomitant rescue of long-term memory deficits. These results are in line with the study of Heneka et al. [16] showing the same effects in APP_swe_/PS1dE9 mice KO for NALP3. It is important to notice that NALP3 KO mice displayed a 50 % decrease in IL-1β production, corresponding to our in vitro situation in ASC+/− astrocytes. It seems, however, that high levels or minimal production of IL-1β, as observed in ASC+/+ and ASC−/− astrocytes could decrease their phagocytic activity.

## Conclusions

Since the first study showing an activation of the NALP3 inflammasome by Aβ and its involvement in tissue damage in AD, others reports confirmed the deleterious effect of inflammasome-mediated inflammation. Here, by targeting a central component of the inflammasome protein complex, we demonstrate a beneficial effect of ASC downregulation on amyloid load and memory performances. We demonstrate in vitro that Aβ is able to activate astrocytic inflammasome. These astrocytes seemed to be potent actors in the clearance of amyloid peptide, and ASC heterozygous cells displayed a higher phagocytic efficiency when activated by Aβ. Thus, targeting inflammasome activation in particular in astrocytes seems to be an interesting strategy to fight against amyloid-induced pathological features observed in an AD mouse model.

## Additional files

Additional file 1:
**Neutralization of CCL3 by a specific antibody.** Astrocytes primed with 1 μg/ml LPS for 3 h were treated with 10 μM Aβ42 for 3 h. Specific antibody against CCL3 was added 45 min after Aβ42. The concentration of CCL3 in culture supernatants was then measured by ECLIA. Results are the mean ± SEM derived from three experiments in duplicate, and statistical differences were determined using a Mann-Whitney test. ****P* < 0.001, ***P* < 0.01, and **P* < 0.05 vs respective controls. Additional file 2:
**Path length (cm) measured during the probe trial in the Morris water maze (MWM).** Mice were let in the MWM for a 1-min free swimming test. Total distances traveled were quantified and showed no difference between all tested groups (15 F− A+/+, 14 F+ A+/+, and 11 F+ A+/−). Additional file 3:
**TNFα varies in inverse proportion to IL-1β.** Astrocytes primed with 1 μg/ml LPS for 3 h were treated with 10 μM Aβ42 for 3 h. The concentration of TNFα or IL-1β released in culture supernatants were then measured by specific ECLIA. Results are the mean ± SEM, and statistical differences were determined using an ANOVA analysis and post Bonferroni multiple comparison test (*n* = 4 in duplicate). ****P* < 0.001, ***P* < 0.01, and **P* < 0.05 (*n* = 4 in duplicate for all genotypes).
