# Supplementary material for: Activation of phagocytic activity in astrocytes by reduced expression of the inflammasome component ASC and its implication in a mouse model of Alzheimer disease
Source: J Neuroinflammation. 2016 Jan 27;13:20. doi: 10.1186/s12974-016-0477-y (PMC4729126; doi:10.1186/s12974-016-0477-y)
Supplement: Additional file 1: — Neutralization of CCL3 by a specific antibody. Astrocytes primed with 1 μg/ml LPS for 3 h were treated with 10 μM Aβ42 for 3 h. Specific antibody against CCL3 was added 45 min after Aβ42. The concentration of CCL3 in culture supernatants was then measured by ECLIA. Results are the mean ± SEM derived from three experiments in duplicate, and statistical differences were determined using a Mann-Whitney test. ***P < 0.001, **P < 0.01, and *P < 0.05 vs respective controls. [file 12974_2016_477_MOESM1_ESM.pdf]

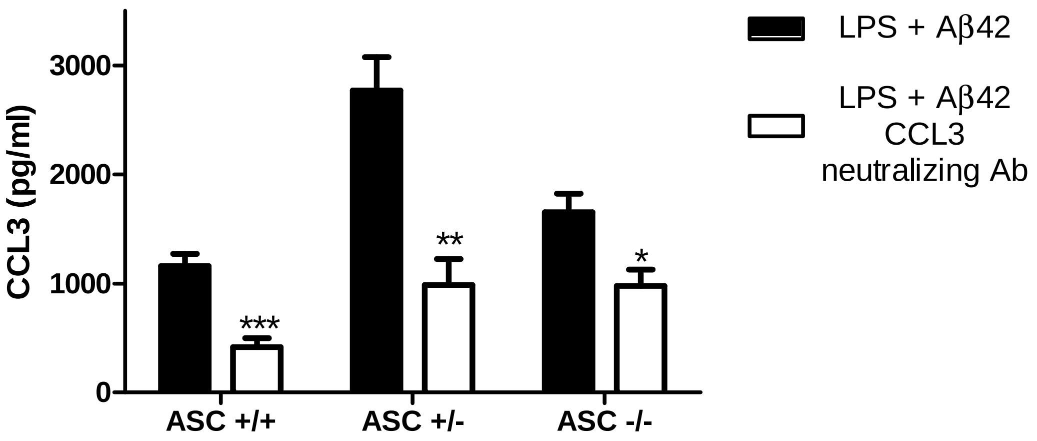

Additional file 1: Neutralization of CCL3 by a specific antibody. Astrocytes primed with 1µg/ml LPS for 3h were treated with 10µM Aβ42 for 3h. Specific antibody against CCL3 was added 45min after Aβ42. The concentration of CCL3 in culture supernatants were then measured by ECLIA. Results are the mean ± SEM derived from 3 experiments in duplicate and statistical differences were determined using a Mann Whitney test. ****P*<0.001, ***P*<0.01 and **P*<0.05 vs respective controls.
