# Supplementary material for: Activation of phagocytic activity in astrocytes by reduced expression of the inflammasome component ASC and its implication in a mouse model of Alzheimer disease
Source: J Neuroinflammation. 2016 Jan 27;13:20. doi: 10.1186/s12974-016-0477-y (PMC4729126; doi:10.1186/s12974-016-0477-y)
Supplement: Additional file 2: — Path length (cm) measured during the probe trial in the Morris water maze (MWM). Mice were let in the MWM for a 1-min free swimming test. Total distances traveled were quantified and showed no difference between all tested groups (15 F− A+/+, 14 F+ A+/+, and 11 F+ A+/−). [file 12974_2016_477_MOESM2_ESM.pdf]

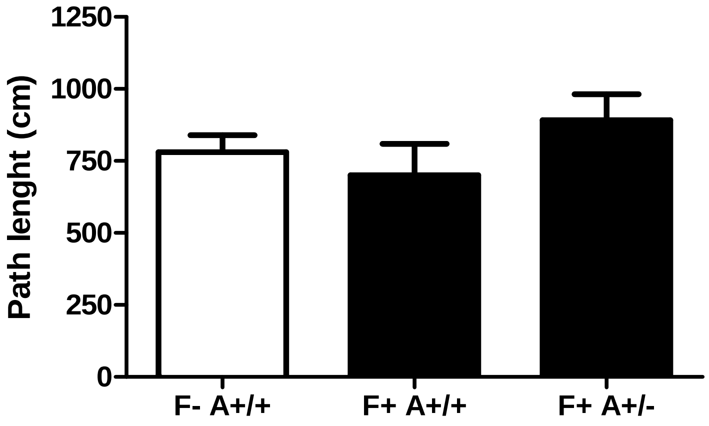

Additional file 2: Path length (cm) measured during the probe trial in the Morris Water Maze (MWM). Mice were let in the MWM for a 1min free swimming test. Total distances travelled were quantified and showed no difference between all tested groups (15 F-A+/+, 14 F+A+/+ and 11 F+A+/-).
