# Supplementary material for: Activation of phagocytic activity in astrocytes by reduced expression of the inflammasome component ASC and its implication in a mouse model of Alzheimer disease
Source: J Neuroinflammation. 2016 Jan 27;13:20. doi: 10.1186/s12974-016-0477-y (PMC4729126; doi:10.1186/s12974-016-0477-y)
Supplement: Additional file 3: — TNFα varies in inverse proportion to IL-1β. Astrocytes primed with 1 μg/ml LPS for 3 h were treated with 10 μM Aβ42 for 3 h. The concentration of TNFα or IL-1β released in culture supernatants were then measured by specific ECLIA. Results are the mean ± SEM, and statistical differences were determined using an ANOVA analysis and post Bonferroni multiple comparison test (n = 4 in duplicate). ***P < 0.001, **P < 0.01, and *P < 0.05 (n = 4 in duplicate for all genotypes). [file 12974_2016_477_MOESM3_ESM.pdf]

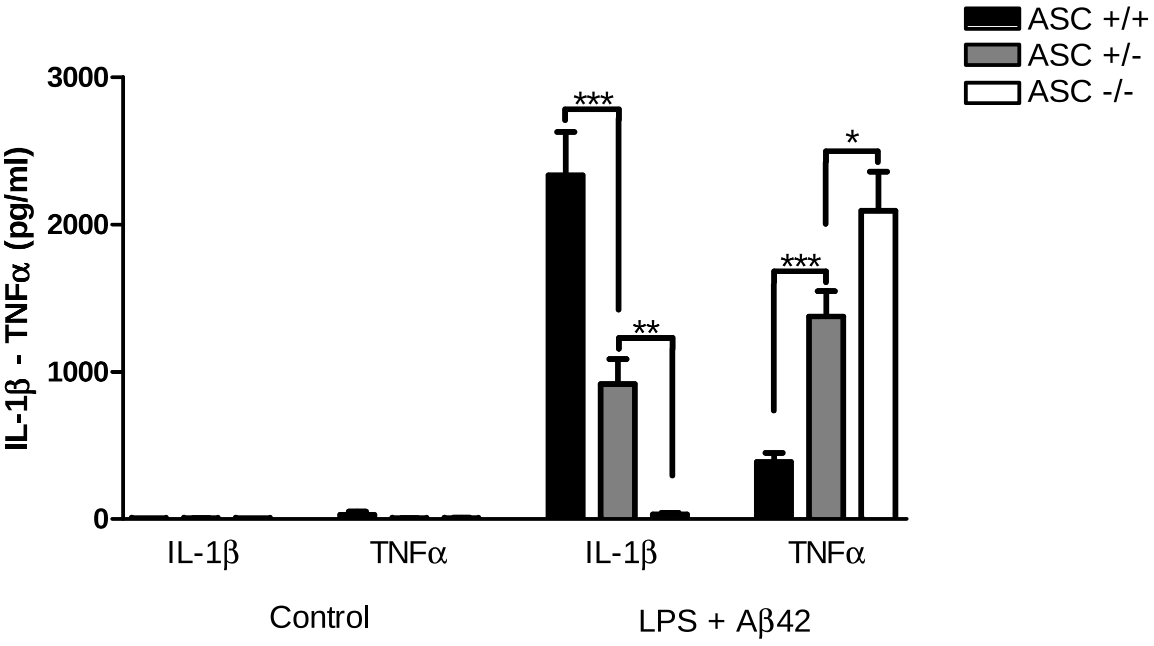

Additional file 3: TNFα varies in inverse proportion to IL-1β. Astrocytes primed with 1µg/ml LPS for 3h were treated with 10µM *Aβ*42 for 3h. The concentration of TNFα or IL-1β released in culture supernatants were then measured by specific ECLIA. Results are the mean ± SEM and statistical differences were determined using an ANOVA analysis and post Bonferroni's Multiple Comparison Test (n=4 in duplicate). ***P<0.001, **P<0.01 and *P<0.05 (n=4 in duplicate for all genotypes).
